# Large-Scale Evaluation of Candidate Genes Identifies Associations between *VEGF* Polymorphisms and Bladder Cancer Risk

**DOI:** 10.1371/journal.pgen.0030029

**Published:** 2007-02-23

**Authors:** Montserrat García-Closas, Núria Malats, Francisco X Real, Meredith Yeager, Robert Welch, Debra Silverman, Manolis Kogevinas, Mustafa Dosemeci, Jonine Figueroa, Nilanjan Chatterjee, Adonina Tardón, Consol Serra, Alfredo Carrato, Reina García-Closas, Cristiane Murta-Nascimento, Nathaniel Rothman, Stephen J Chanock

**Affiliations:** 1 Division of Cancer Epidemiology and Genetics, National Cancer Institute, Department of Health and Human Services, Bethesda, Maryland, United States of America; 2 Center for Research in Environmental Epidemiology, Municipal Institute of Medical Research (IMIM), Barcelona Spain; 3 Cellular and Molecular Biology Research Unit, Municipal Institute of Medical Research (IMIM), Barcelona, Spain; 4 Department of Experimental and Health Sciences, Universitat Pompeu Fabra, Barcelona, Spain; 5 Core Genotype Facility at the Advanced Technology Center of the National Cancer Institute, Department of Health and Human Services, Bethesda, Maryland, United States of America; 6 Department of Social Medicine, Medical School, University of Crete, Heraklion, Greece; 7 Department of Preventive Medicine and Public Health, Universidad de Oviedo, Oviedo, Spain; 8 Unit of Research in Occupational Health, Department of Experimental and Health Sciences, Universitat Pompeu Fabra, Barcelona, Spain; Corporació Parc Taulí, Sabadell, Spain; 9 Department of Medical Oncology, Hospital General de Elche, Elche, Spain; 10 Department of Preventive Medicine, Hospital Universitario de Canarias, La Laguna, Spain; University of Michigan, United States of America

## Abstract

Common genetic variation could alter the risk for developing bladder cancer. We conducted a large-scale evaluation of single nucleotide polymorphisms (SNPs) in candidate genes for cancer to identify common variants that influence bladder cancer risk. An Illumina GoldenGate assay was used to genotype 1,433 SNPs within or near 386 genes in 1,086 cases and 1,033 controls in Spain. The most significant finding was in the 5′ UTR of *VEGF* (rs25648, *p* for likelihood ratio test, 2 degrees of freedom = 1 × 10^−5^). To further investigate the region, we analyzed 29 additional SNPs in *VEGF,* selected to saturate the promoter and 5′ UTR and to tag common genetic variation in this gene. Three additional SNPs in the promoter region (rs833052, rs1109324, and rs1547651) were associated with increased risk for bladder cancer: odds ratio (95% confidence interval): 2.52 (1.06–5.97), 2.74 (1.26–5.98), and 3.02 (1.36–6.63), respectively; and a polymorphism in intron 2 (rs3024994) was associated with reduced risk: 0.65 (0.46–0.91). Two of the promoter SNPs and the intron 2 SNP showed linkage disequilibrium with rs25648. Haplotype analyses revealed three blocks of linkage disequilibrium with significant associations for two blocks including the promoter and 5′ UTR (global *p* = 0.02 and 0.009, respectively). These findings are biologically plausible since *VEGF* is critical in angiogenesis, which is important for tumor growth, its elevated expression in bladder tumors correlates with tumor progression, and specific 5′ UTR haplotypes have been shown to influence promoter activity. Associations between bladder cancer risk and other genes in this report were not robust based on false discovery rate calculations. In conclusion, this large-scale evaluation of candidate cancer genes has identified common genetic variants in the regulatory regions of *VEGF* that could be associated with bladder cancer risk.

## Author Summary

This article reports findings from a large-scale evaluation of common variation in candidate genes for cancer to identify variants that influence bladder cancer risk. We first evaluated 1,433 common variants within or near 386 genes in a large case-control study in Spain. The most significant finding was the gene coding for the vascular endothelial growth factor *(VEGF).* To further investigate this finding, we identified markers that captured most common variation in the whole gene. Analyses indicated that variants in regulatory regions of *VEGF* could modify the risk for developing bladder cancer. This association is biologically plausible since *VEGF* is critical for the growth of new blood vessels, which is important for tumor development, and its elevated expression in bladder tumors correlates with tumor progression. Future studies are required to confirm these findings, as well as to investigate the mechanisms for the observed associations.

## Introduction

Bladder cancer is primarily a sporadic disease, and environmental factors such as tobacco smoking and occupational exposure to aromatic amines have been established as strong determinants of risk [1]. A moderate familial component has been demonstrated for bladder cancer, but so far no high-penetrance mutations have been described [1]. However, there is strong evidence for the influence of common genetic variants on bladder cancer risk. Most notably, large studies have demonstrated associations with each of the *NAT2* and *GSTM1* genotypes and a probable interaction between smoking and *NAT2* genotype [2]. Specifically, the *GSTM1* null genotype increases the overall risk of bladder cancer; while the *NAT2* slow acetylator genotype appears to increase risk particularly among cigarette smokers [2]. In this context, we hypothesized that a large-scale effort to screen common variants in candidate cancer genes could identify additional bladder cancer susceptibility genes.

The recent development of highly multiplexed single nucleotide polymorphism (SNP) genotyping assays has resulted in an opportunity to screen candidate genetic variants in an affordable, high-throughput manner in epidemiological studies. We used a GoldenGate assay by Illumina targeted to analyze over 1,500 SNPs in selected candidate cancer genes in order to identify bladder cancer susceptibility genes using samples collected in a large case-control study of bladder cancer in Spain. Because this was one of the first epidemiological studies using this highly multiplexed technology, we performed a detailed analysis of data quality. All SNPs chosen for this platform were drawn from the SNP500Cancer public database (http://snp500cancer.nci.nih.gov), which includes genes or specific genetic variants that could be important in cancer and have been re-sequenced in 102 individuals [3].

## Results

We obtained high-quality genotype calls from 1,433 SNP assays in or near 386 genes involved in cancer-related pathways, with a median of two SNPs per gene (range: 1–37 SNPs per gene). About half (51%) of these SNPs were located in introns, 32% in exons, 12% in promoter regions, and 5% in 3′ of stop codon (STP). For SNPs located in exons, 5% were in 5′ UTRs, 68% in coding regions (approximately half were synonymous and half non-synonymous changes) and 27% in 3′ UTRs. The median (range) minor allele frequency (MAF) among controls was 0.24 (0.02–0.50).

The global genotype completion for study samples was ≥99%. Genotype concordance in 69 blinded duplicate blood DNA pairs was ≥99%. Of the 1,433 SNPs in the GoldenGate assay, 72 SNPs had been previously typed using other genotyping platforms on 2,256 blood DNA samples from study participants, and 31 of these SNPs had been previously genotyped on 50 buccal DNA samples from the study. Genotype concordance between the GoldenGate and other platforms (primarily TaqMan) was ≥98%. About 5% (79/1433) of genotype assays had a significant (*p* < 0.05) departure from Hardy-Weinberg equilibrium, which is consistent with what would be expected by chance.

Gene-based (Table 1) and SNP-based (Table S1) analyses showed promising associations (i.e., *p*-value for trend or likelihood ratio test [LRT]; 2 degrees of freedom [df] <0.01) with bladder cancer risk for 19 genes: *VEGF, STK11, CYP1B1, ZNF350, PTH, GHR, CASP9, PLA2G6, GSTA4, ROS1, RB1CC1, TERT, XRCC4, FZD7, CETP, CYP24A1, LIPC, ESR1,* and *HSD17B4.* In addition, *ARHGDIB, SHBG, GPX4,* and *STAT1* showed significant associations with risk according to the LRT (2 df); however, estimates for heterozygous and homozygous variants showed associations in opposite directions (Table S2).

**Table 1:**
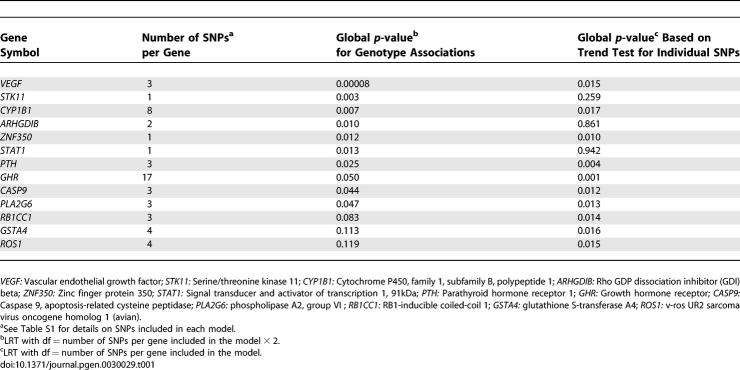
Global Gene *p*-values for Significant (*p*-value ≤ 0.01) Associations between Bladder Cancer and Common Genetic Variation in Selected Candidate Genes among the 1,086 Cases and 1,033 Controls in the Spanish Bladder Cancer Study

The most significant association with bladder cancer risk according to gene-based analyses was observed for the vascular endothelial growth factor *(VEGF)* gene (Table 1). We evaluated these findings using the false discovery rate (FDR) approach described in Materials and Methods. FDR values for the *VEGF* association with bladder cancer risk, taking into account all 386 genes evaluated in this report, was 0.032 based on the global *p*-values from LRTs (2 df) performed for each gene. The next lowest FDR value was 0.56 for *STK11,* indicating that the associations for other genes were not robust. We also calculated FDR values for the 386 trend tests performed for each gene, and the lowest FDR value was 0.51 for *GHR,* with a value of 0.64 for *VEGF.*

Individual SNP analyses showed the strongest association for a variant allele in the 5′ UTR of *VEGF* (rs25648, RefSNP accession number assigned by dbSNP, http://www.ncbi.nlm.nih.gov/projects/SNP). The MAF for this SNP among the control population was 0.14, and the odds ratio (OR) (95% confidence interval [CI]) for heterozygote and homozygote variant genotypes compared to the common homozygote genotype was 1.12 (0.91–1.37) and 5.11 (2.33–11.20), respectively; *p*-values for LRT (2 df) = 1 × 10^−5^ and for trend test = 0.002 (Table 2). The observed frequency for the homozygote variant genotype was lower than expected under Hardy-Weinberg equilibrium in the control population (0.8% observed versus 1.9% expected, *p*-value = 0.002), while genotype completion and concordance for this SNP were measured at 100%. To explore the impact of the observed departure on estimates of relative risk, we re-estimated ORs (95% CIs) assuming Hardy-Weinberg equilibrium [4]. Estimates for heterozygote and homozygote variant genotypes assuming Hardy-Weinberg equilibrium were 1.23 (1.02–1.48) *p*-value = 0.03 and 2.20 (1.48–3.28) *p*-value = 0.00009, respectively.

**Table 2:**
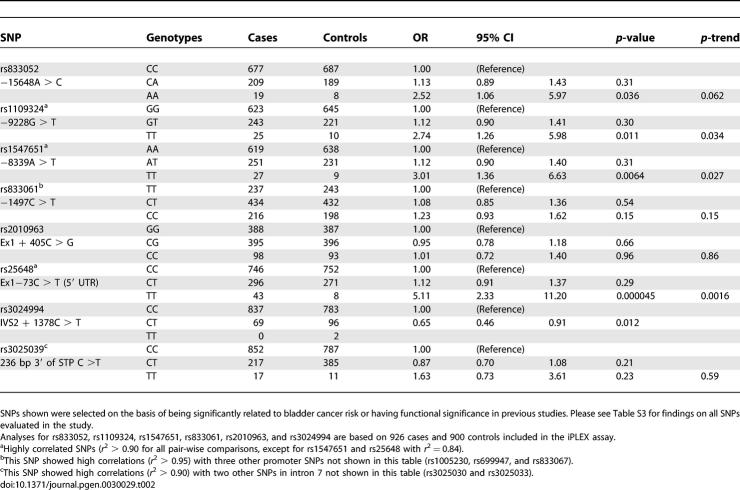
Association between Selected *VEGF* Polymorphisms with Bladder Cancer Risk among 1,086 Cases and 1,033 Controls in the Spanish Bladder Cancer Study

We performed additional genotyping for 29 SNPs in *VEGF* (including the three SNPs previously genotyped) in an effort to dissect the locus to follow-up findings from our exploratory analysis described above. Two of these SNPs showed low genotypic variation in this population (no variants were observed for rs3024989, and three controls and no cases were heterozygote for rs9367173). The concordance for the three *VEGF* SNPs previously genotyped in the GoldenGate assay (rs1005230, rs25648, and rs3025039) was 100%. Genotype completion and concordance rates for all *VEGF* SNPs exceeded 99%, and all but rs25648 (*p* = 0.002) were in Hardy-Weinberg equilibrium in controls. Analyses showed significant associations with three additional SNPs located in the promoter region of *VEGF* (rs833052, rs1109324, and rs1547651) and one SNP in intron 2 (rs3024994) (Table 2). However, the association for the rs833052 promoter SNP was only borderline significant. Two of these SNPs (rs1109324 and rs1547651) were in strong linkage disequilibrium (LD) with the 5′ UTR SNP (D′ ≥ 0.94 and *r* ^2^ ≥ 0.84). None of the other SNPs showed significant associations with bladder cancer risk (see Tables 2, S3, and S4 for more details).

We evaluated interactions between the *VEGF* SNPs significantly associated with bladder cancer risk and other determinants of risk (i.e., age, gender, smoking status, family history of cancer in at least one first-degree relative, and *NAT2* and *GSTM1* genotypes). Analyses suggested stronger associations for the two correlated SNPs in the *VEGF* promoter (rs1109324 and rs1547651) among subjects with a family history of cancer (*p*-value for heterogeneity = 0.035 and 0.036, respectively; Table S5). We also observed a stronger association for the 5′ UTR SNP (rs25648) among subjects with the *GSTM1* null genotype (*p*-value for heterogeneity = 0.031; Table S5). However, these findings need to be interpreted with caution given the number of interactions evaluated.

**Figure 1:**
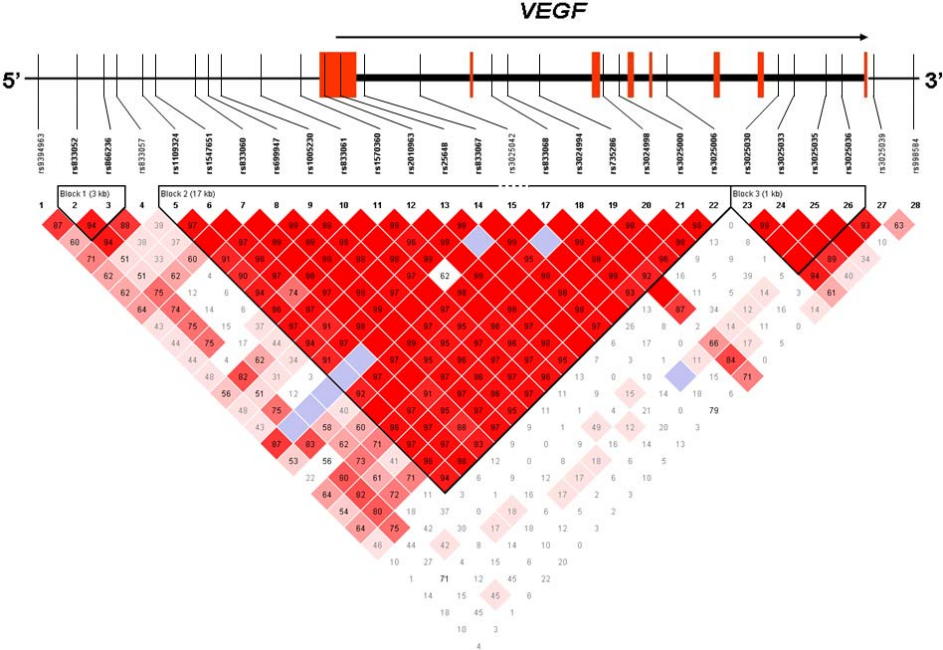
Gene Map and LD Plot of *VEGF* Gene Color scheme is based on D′ and logarithm of the odds of linkage (LOD) score values: white D′ < 1 and LOD < 2, blue D′ = 1 and LOD < 2, shades of pink/red: D′ < 1 and LOD ≥ 2, and bright red D′ = 1 and LOD ≥ 2. Numbers in squares are D′ values (values of 1.0 are not shown). Block definition is based on the Gabriel et al. method [34]. Two (rs3024989 and rs367173) of the 29 SNPs determined are not shown because of low variation in this population. Red rectangles in the gene map represent exons.

**Figure 2:**
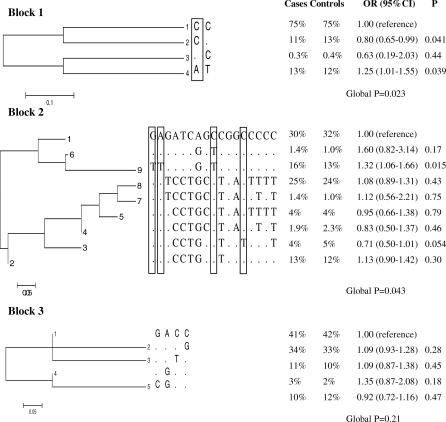
Phylogenetic Trees for *VEGF* Haplotypes and Association with Bladder Cancer Risk among 926 Cases and 900 Controls with DNA in the iPLEX Assay, Spanish Bladder Cancer Study See Figure 1 for block definitions. Of the 29 *VEGF* SNPs determined, two had low genotypic variation in our population; therefore, haplotype analyses were based on the remaining 27 SNPs. Polymorphic bases are in 5′ to 3′ order: Block 1(**rs833052** and rs866236); Block 2 (**rs1109324**, **rs1547651**, rs833060, rs699947, rs1005230, rs833061, rs1570360, rs2010963, **rs25648**, rs833067, rs3025042, rs833068, **rs3024994**, rs735286, rs3024998, rs3025000, and rs3025006); and Block 3 (rs3025030, rs3025033, rs3025035, and rs3025036). Bolded rs numbers are for individual SNPs significantly associated with bladder cancer risk. Eleven cases and 13 controls with missing data on more than 15 of the 17 SNPs in Block 2 were excluded from haplotype analyses because their inclusion resulted in lack of convergence. Nucleotide changes significantly associated with risk in the individual genotype analyses are shown in boxes. The most common haplotye is the reference category. Haplotypes with the common variant for each individual SNP are CC for Block 1, GAGCCGTGCTGGCCCCC for Block 2, and GACC for Block 3.

Haplotype analyses were based on 27 *VEGF* SNPs (of the 29 SNPs determined, two were excluded because of low genotypic variation). Of the three large blocks defined by LD in our control population (Figure 1), we observed significant associations between haplotypes and risk for bladder cancer in two LD blocks, including the promoter and 5′ UTR (global *p* = 0.023 and 0.043 for blocks 1 and 2, respectively; Figure 2). Consistent with individual SNP analyses, the AT haplotype in block 1 carrying the variant allele for rs833052 was associated with increased bladder cancer risk; however, the CT haplotype was related to decreased bladder cancer risk, which was not predicted by individual SNP analyses. Both the individual SNP and haplotype associations were only of borderline significance and thus could be due to chance.

Of the nine observed haplotypes in block 2, only one (GAGCCGTGCTGGTCCCT) carried the variant in intron 2 (rs3024994) that was individually associated with reduced risk (Figure 2). Consistent with SNP analyses, this haplotype was also associated with a reduction in risk. Two other haplotypes carried at least one variant for three correlated SNPs individually associated with risk (rs1109324, rs1547651, and rs25648). Both haplotypes were associated with increases in risk, although the association for the haplotye carrying only the rs25648 variant (GAGATGCGTCGGCCCCC) was not significant, possibly due to its low frequency in the population (1.0% of controls) (Figure 2). Therefore, haplotype analyses cannot help distinguish which of the three correlated SNPs is most important in determining risk.

## Discussion

An exploratory analysis of 1,433 SNPs in or near 386 genes involved in cancer-related pathways using the GoldenGate assay led to the identification of novel associations for several promising genes, the most notable finding, a 5′ UTR SNP in *VEGF.* Subsequent analyses that captured nearly all common variants in this gene showed additional associations with SNPs in the promoter and intron 2, providing further evidence for the importance of variation in regulatory elements of *VEGF* and bladder cancer risk.

The association of common genetic variation in *VEGF* with bladder cancer risk is biologically plausible for several reasons: (1) *VEGF* has been identified as a critical factor in angiogenesis required for tumor growth, (2) *VEGF* expression in bladder tumors has been related to tumor progression [5], and (3) in vitro studies have suggested that common haplotypes in the 5′ region of *VEGF* alter gene expression [6]. A large block of LD that extended from the promoter to intron 5 included the 5′ UTR SNP (rs25648) that demonstrated the strongest association with bladder cancer risk in our initial screen. This SNP showed a departure from Hardy-Weinberg equilibrium in controls (*p*-value = 0.002), which was not observed for two correlated SNPs in the promoter region (rs1109324 and rs1547651; *r* ^2^ > 0.80 between the 5′ UTR and promoter SNPs) also associated with risk. We did not observe more deviations from Hardy-Weinberg equilibrium than expected by chance in our control population, the deviation for rs25648 was not observed in other Caucasian populations [7] (http://snp500cancer.nci.nih.gov), and quality control samples did not show evidence for genotype errors; therefore, this departure is likely to have occurred by chance. The observed magnitude of the association for rs25648 was larger than for the two promoter SNPs; however, this difference was less apparent after re-estimation of ORs assuming Hardy-Weinberg equilibrium, suggesting that it might have occurred by chance.

Carriers of the 5′ UTR SNP (rs25648) have been found to have increased VEGF mRNA levels in adenocarcinoma tissues of patients with colorectal adenocarcinomas [6]. Although there are no functional studies of the two promoter SNPs associated with bladder cancer risk in our study population, previous studies have shown that variant genotypes or haplotypes falling in the same block of LD (rs699947, rs1570360, rs833061, and rs2010963) were associated with (1) higher induced gene expression from hypoxia in transient transfection assays [8]; (2) higher VEGF production in peripheral blood mononuclear cells [9,10]; (3) increased promoter activity and responsiveness to phorbol esters in breast cancer cell lines [11]; and (4) tumor aggressiveness in breast cancer patients [12]. Finally, the SNP in intron 2 (rs3024994) associated with reduced risk fell in the same block of LD but showed low correlation with other SNPs, and there are no published studies on its functional significance.

Only one SNP in *VEGF* (rs699947) has been previously evaluated in relation to bladder cancer risk in a small study of 153 bladder cancer patients and 153 controls in South Korea [13]. Consistent with our results, this study found no association between this SNP and bladder cancer risk. A SNP in 3′ of STP (rs3025039) has been associated with decreased plasma levels of VEGF and decreased breast cancer risk [14]; however, neither this nor other SNPs in LD were associated with bladder cancer risk in our study.

We also observed promising associations with bladder cancer risk for other genes involved in carcinogenesis pathways. However, based on FDR calculations, taking into account all genes evaluated in this report, the additional associations were not robust and thus should be pursued in additional study populations.

To the best of our knowledge, this is the first large-scale evaluation of candidate genes in bladder cancer using highly multiplexed technologies. Our data demonstrate that these technologies provide high quality data and that they can be useful in identifying genetic susceptibly factors. In particular, we provide reasonable evidence for an association between common variants in the promoter and 5′ UTR of *VEGF* and bladder cancer risk. Further work is required to replicate the findings in other populations and to identify the potential causal variant by more detailed genetic mapping, including sequencing and functional characterization of variants.

## Materials and Methods

### Study population.

The study population has been previously described [2]. Briefly, cases were patients participating in the Spanish Bladder Cancer Study diagnosed with histologically confirmed bladder carcinoma in 1998–2001, aged 21–80 y (mean [sd] = 66 [10] y), of which 87% were males. Controls were selected from patients admitted to participating hospitals for diagnoses believed to be unrelated to the exposures of interest, individually matched to the cases by age at interview within 5-y categories, gender, ethnicity, and region. Demographic and risk factor information was collected at the hospitals using computer-assisted personal interviews. A total of 1,219 cases (84% of eligible cases) and 1,271 controls (88% of eligible controls) agreed to participate in the study and were interviewed. Of these, 1,188 (97%) cases and 1,173 (92%) controls provided a blood or buccal cell sample for DNA extraction. Adequate amounts of DNA for genotyping were available from 1,116 cases (including eight from buccal cells) and 1,043 controls (including 36 from buccal cells). Further exclusions were made to reduce heterogeneity (cases with nontransitional histology and nonwhite subjects), or because of DNA contamination or lack of information on smoking status. After exclusions, the available samples for genotype analysis were 1,086 cases and 1,033 controls.

We obtained informed consent from potential participants in accordance with the National Cancer Institute and local institutional review boards.

### Genotyping.

A GoldenGate assay (Illumina, http://www.illumina.com) was developed using SNPs in the SNP500Cancer project (http://snp500cancer.nci.nih.gov) with previous re-sequence analysis and plausible evidence that the gene is related to carcinogenic processes [3]. SNP selection favored nonsynonymous SNPs, those previously evaluated in relation to cancer risk, or those with evidence for functional significance. The GoldenGate assay was designed to examine 1,536 SNPs based on an initial screen of 3,072 SNPs drawn from the SNP500Cancer database (November 2004) and subsequently analyzed in the unrelated HapMap Centre d'Etude du Polymorphisme Humain (CEPH) Utah samples. Of the 1,536 assays chosen for this study, 103 were dropped from the analysis because of low MAF or assay problems. Thus, we obtained data on 1,433 SNPs in or near 386 genes (Table S1). DNA samples from cases and controls were randomly sorted, including 69 duplicated DNA samples for genotyping quality control.

Based on our primary analysis that showed the strongest association of bladder cancer with an SNP in *VEGF,* we performed a comprehensive evaluation of common variation in this gene. We initially selected 31 SNPs spanning 20 kb 5′ of the start of transcription to 10 kb 3′ of the end of exon 8 of the *VEGF* gene using the following methods: (1) 15 tag SNPs were chosen based on the aggressive tagging algorithm [15] (*r* ^2^ ≥ 0.80, MAF ≥ 0.05) using genotype data from the unrelated HapMap CEPH Utah individuals; (2) 16 SNPs from the Single Nucleotide Polymorphism database were added as “fill-in” to ensure the inclusion of an SNP every 2–5 kb across the region, particularly in the 5′ region. iPLEX (Sequenom, http://www.sequenom.com) assays were designed and optimized with the SNP500Cancer set of 102 individuals. Two SNPs were dropped because of design and performance problems. Out of 29, 28 assays were optimized on iPLEX and one SNP (rs699947) that could not be included was analyzed using TaqMan (Applied Biosystems, http://www.appliedbiosystems.com). Because of restricted amounts of DNA available and poor assay performance for a small subset of samples and exclusions for data analyses described earlier (cases with nontransitional histology, nonwhite subjects, and lack of information on smoking status), a total of 926 cases and 900 controls were included in the analyses.

### Statistical analysis.

For each individual SNP, we estimated OR and 95% CI using logistic regression models adjusting for gender, age at interview in 5-y categories, region, and smoking status (never, occasional, former, and current; see [2] for details on the definition of these variables). The association between individual SNPs and bladder cancer risk was tested using a 2-df LRT and a linear trend test assuming a dose response with increasing number of variant alleles. ORs and 95% CIs “per variant allele” were estimated under the latter assumption (i.e., coding genotypes as 0, 1, and 2 depending on the number of variant alleles). Heterogeneity of genotype ORs among groups of subjects defined by age, gender, smoking status, family history of cancer in at least one first-degree relative, and *NAT2* and *GSTM1* genotypes (see [2] for details on the definition of these variables) were evaluated by introducing interaction terms in logistic regression models.

The 1,433 individual SNPs evaluated were located within or near 386 candidate genes. We performed two gene-based tests for association: (1) an LRT for each gene comparing models with and without terms for heterozygous and homozygous variant genotypes for each SNP in a given gene (df = 2 × number of SNPs per gene); (2) an LRT for each gene comparing models with and without terms for each SNP (genotypes coded as 0, 1, and 2) in a given gene (df = number of SNPs per gene). For highly correlated SNPs (*r* ^2^ > 0.90) within a gene, only one of the SNPs was included in the model to avoid collinearity problems (Table S1).

Haplotype frequencies, ORs, and 95% CIs for genes showing blocks of LD were estimated using HaploStats (http://mayoresearch.mayo.edu/mayo/research/biostat/schaid.cfm). This program reconstructs haplotypes and estimates ORs simultaneously based on a suitable Expectation-Maximization algorithm [16,17].

We used the method described by J. Chen and N. Chatterjee to obtain estimates and *p*-values for genotype associations assuming Hardy-Weinberg equilibrium in the control population [4]. Phylogenetic trees (neighbor joining [18]) were constructed using MEGA 3.1 [19] (http://www.megasoftware.net) to assess nucleotide similarity of different haplotypes.

We evaluated the robustness of our results using the FDR. FDR is the expected ratio of erroneous rejections of the null hypothesis to the total number of rejected hypothesis among all the genes or SNPs analyzed in this report. Rather than using an arbitrary threshold FDR value, we report the values for the most significant associations to allow the reader to evaluate the robustness of our findings. The Benjamini and Hochberg method [20] was used to calculate FDR values using “multtest” package in the R project for statistical analyses (http://www.r-project.org). Unless otherwise specified, statistical analyses were performed with STATA Version 8.2, Special Edition (STATA Corporation, http://www.stata.com).

## Supporting Information

Table S1 List of SNPs Included in the SNP500 Illumina GoldenGate Assay, Spanish Bladder Cancer Study (1.2 MB DOC).

Table S2 SNP-Based Analyses: Association between Bladder Cancer Risk and SNPs with a *p*-value from a Trend Test or a 2-df LRT ≤ 0.01 in the Spanish Bladder Cancer Study (34 KB DOC).

Table S3 Association between Common Variants in *VEGF* and Bladder Cancer Risk in the Spanish Bladder Cancer Study (233 KB DOC).

Table S4 Genotype Cell Counts for Cases and Controls (19 KB XLS).

Table S5 Modification of the Association between Selected Variants in *VEGF* and Bladder Cancer Risk by Age, Gender, Smoking Status, Family History of Cancer, *NAT2* and *GSTM1* Genotypes (173 KB DOC).

## 

### Accession Numbers

The accession numbers for the Entrez Gene (http://www.ncbi.nlm.nih.gov/entrez) genes discussed in this paper are *ARHGDIB* (397), *CASP9* (842), *CETP* (1071), *CYP1B1* (1545), *CYP24A1* (1591), *ESR1* (2099), *FZD7* (8324), *GHR* (2690), *GPX4* (2879), *GSTA4* (2941), *HSD174* (3295), *LIPC* (3990), *PLA2G6* (8398), *PTH* (5741), *RB1CC1* (9821), *ROS1* (6098), *SHBG* (6462), *STAT1* (6772), *STK11* (6794), *TERT* (7015), *VEGF* (7422), *XRCC4* (7518), and *ZNF350* (59348).

## Abbreviations

CI: confidence interval
df: degrees of freedom
FDR: false discovery rate
LD: linkage disequilibrium
LRT: likelihood ratio test
MAF: minor allele frequency
OR: odds ratio
SNP: single nucleotide polymorphism
STP: stop codon
