# Supplementary material for: Large-Scale Evaluation of Candidate Genes Identifies Associations between VEGF Polymorphisms and Bladder Cancer Risk
Source: PLoS Genet. 2007 Feb 23;3(2):e29. doi: 10.1371/journal.pgen.0030029 (PMC1802828; doi:10.1371/journal.pgen.0030029)
Supplement: Table S1 — (1.2 MB DOC). [file pgen.0030029.st001.doc]

Supplementary Table 1: List of SNPs included in the SNP500 Illumina Golden Gate Assay, Spanish Bladder Cancer Study.

| **Gene symbol** | **SNPs included in analyses rs number*** | **SNPs excluded from analyses** rs number** |
| --- | --- | --- |
| *Genes with a single SNP* |  |  |
| *ABCA5* | 15886 |  |
| *AKR1A1* | 2088102 |  |
| *AKR1C4* | 3829125 |  |
| *AKT1* | 2498799 |  |
| *ALDH2* | 2238151 |  |
| *ALOX12* | 1126667 |  |
| *ANKK1* | 1800497 |  |
| *APOE* | 440446 |  |
| *ASE-1* | 3212986 |  |
| *BIRC2* | 1943781 |  |
| *BPI* | 1131847 |  |
| *C14ORF25 (FOXA1)* | FOXA1-41 |  |
| *CASP10* | 3900115 |  |
| *CBR3* | 881712 |  |
| *CD14* | 2569190 |  |
| *CD4* | 3213427 |  |
| *CDC25A* | 936426 |  |
| *CDC25B* | 910656 |  |
| *CDC25C* | 1042124 |  |
| *CDK7* | 2972388 |  |
| *CDKN1B* | 7330 |  |
| *CDKN1C* | 431222 |  |
| *CSF2* | 25882 |  |
| *CTSB* | 1065712 |  |
| *CTSH* | 3129 |  |
| *CYP2C19* | 4244285 | 4986894 |
| *CYP2D6* | 2854741 |  |
| *CYP3A4* | CYP3A4-57 |  |
| *CYP3A7* | 12360 |  |
| *CYBB* | 5964149 | 4422908 |
|  |  | 5964125 |
|  |  | 6610650 |
|  |  | 5964151 |
| *DRD1* | 5326 |  |
| *ENG* | 1330684 |  |
| *ENPP1* | 1044582 |  |
| *EPHX2* | 1126452 |  |
| *ERBB2* | 1810132 |  |
| *ERCC6* | 2228529 | 2228527 |
| *FASLG* | 929087 |  |
| *FUT2* | 603985 |  |
| *GC* | 7041 |  |
| *GRPR* | 4986945 | 4986946 |
| *HADHA* | 1049987 | 7260 |
|  |  | 2289019 |
| *HIF1AN* | 2295780 |  |
| *HMGCR* | 2241402 | 2303151 |
| *HSPB8* | 11038 |  |
| *HSU24186* | 2642219 |  |
| *HUS1* | 2242478 | 1056663 |
| *IFNG* | 1861494 |  |
| *IFNGR2* | 1059293 |  |
| *IGFBP1* | 4619 |  |
| *IGFBP3* | 2471551 |  |
| *IL12A* | 582537 |  |
| *IL3* | 40401 |  |
| *IL6* | 1800797 | 1800795 |
| *IL6R* | 8192284 |  |
| *IL8RA* | 2854386 |  |
| *IRF1* | 839 | 9282763 |
| *JTV1* | 2009115 |  |
| *KRT23* | 2269858 |  |
| *LEP* | 2167270 |  |
| *LIG3* | 1052536 |  |
| *LIG4* | 1805386 |  |
| *LMOD1* | 2820312 |  |
| *MAOA* | 6323 |  |
| *MBD4* | 140696 |  |
| *MDM2* | 769412 |  |
| *MEST* | 2072574 |  |
| *METTL1* | 703842 | 2072052 |
| *MPDU1* | 4227 |  |
| *MPO* | 2071409 |  |
| *MTHFD2* | 1667627 |  |
| *MYC* | 3891248 |  |
| *MYNN* | 1317082 |  |
| *NUBP2* | 1065663 | 344357 |
|  |  | 344360 |
| *P2RX7* | 3751144 |  |
| *PHB* | 4987082 |  |
| *PLA2G2A* | 2236771 |  |
| *PLK1* | 40076 |  |
| *POLD1* | 1726787 |  |
| *PTGS1* | 5788 |  |
| *RAC1* | 2303364 |  |
| *RAD54L* | 1048771 |  |
| *RAG1* | 2227973 |  |
| *RGS5* | 15049 |  |
| *SEC14L2* | 1010324 | 2267154 |
|  |  | 2267155 |
| *SELE* | 5361 |  |
| *SEP15* | 540049 | 5845 |
| *SEPT2* | 7568 |  |
| *SLC2A1* | 1770810 |  |
| *SLC2A4* | 5435 |  |
| *SLC30A1* | 2278651 |  |
| *SLC30A4* | 1153829 |  |
| *SLC6A18* | SLC6A18-13 |  |
| *SOD1* | 2070424 |  |
| *SOD3* | 2855262 |  |
| *SSTR3* | 229569 | 86582 |
| *STAT1* | 2066804 |  |
| *STK11* | 741764 |  |
| *SULT1A2* | 3194168 |  |
| *TFF1* | 2839488 |  |
| *TFF3* | 2236705 |  |
| *TFRC* | 3817672 |  |
| *TNFRSF1A* | 887477 |  |
| *TYR* | 1800422 | 1393350 |
| *UGT1A4* | 1042640 |  |
| *XPA* | 1800975 |  |
| *XRCC1* | 25487 |  |
| *ZFPM1* | 904797 |  |
| *ZNF230* | 12753 |  |
| *ZNF350* | 2278414 | 2278415 |
|  |  | 4988334 |
| *Genes with more than one SNP* |  |  |
| *ABCA1* | 2230806 | 7031748 |
|  | 4149313 |  |
|  | 2777801 |  |
|  | 2230808 |  |
|  | 2297404 |  |
| *ABCA6* | 9282552 |  |
|  | 9282553 |  |
| *ABCA7* | 3764651 |  |
|  | 3752241 |  |
| *ABCB1* | 2235074 |  |
|  | 9282564 |  |
|  | 1211152 |  |
| *ABCB11* | 3770603 |  |
|  | 853785 |  |
| *ABCC2* | 717620 |  |
|  | 2273697 |  |
|  | 3740074 |  |
|  | ABCC2-10 |  |
| *ABCC4* | 3765535 |  |
|  | 2274406 |  |
| *ABCG8* | 9282572 | 9282575 |
|  | 6544718 |  |
| *ADH1C* | 698 |  |
|  | 283411 |  |
|  | 2009181 |  |
|  | 17526590 |  |
| *AHR* | 2066853 |  |
|  | 2074113 |  |
|  | 7796976 |  |
| *AHRR* | 10078 |  |
|  | AHRR-02 |  |
| *AKR1C3* | 12529 | 1937845 |
|  | 2275928 | 2245191 |
|  | 10904422 | 1937920 |
|  | 7070041 | 11252937 |
|  | 28942669 | 3763676 |
|  | 28943575 |  |
|  | 17134288 |  |
|  | 10795241 |  |
|  | 6601899 |  |
|  | 7921327 |  |
| *ALAD* | 1139488 |  |
|  | 1805313 |  |
|  | 8177806 |  |
| *ALDH1L1* | 2305230 |  |
|  | 1127717 |  |
|  | 9282690 |  |
| *ALOX5* | 4987105 | 4986832 |
|  | 2029253 |  |
|  | 1369214 |  |
|  | 892691 |  |
|  | 2242332 |  |
|  | 1565097 |  |
| *ALOX15* | 2664593 |  |
|  | 7220870 |  |
| *AMACR* | 2278008 | 10941112 |
|  | 34677 |  |
|  | 34689 |  |
|  | 3195676 |  |
|  | 6863657 |  |
|  | 840409 |  |
| *APAF1* | 2278361 |  |
|  | 2288729 |  |
|  | 1007573 |  |
|  | 1866477 |  |
| *APC* | 41115 | 866006 |
|  | 2229992 |  |
|  | 459552 |  |
|  | 2909786 |  |
| *APEX1* | 3136820 |  |
|  | APEX1-09 |  |
|  | 1760944 |  |
| *APOA2* | 5082 |  |
|  | 6413453 |  |
|  | 5085 |  |
| *APOA4* | apoa4_02 |  |
|  | apoa4_07 |  |
| *APOB* | 1042034 |  |
|  | 1367117 |  |
|  | 1800481 |  |
|  | 1469513 |  |
|  | 3791981 |  |
| *AR* | 1204038 |  |
|  | 1337080 |  |
|  | 1337082 |  |
|  | 2361634 |  |
| *ARHGDIB* | 921 |  |
|  | 2075267 |  |
| *ARNT* | 2256355 | 2228099 |
|  | 7517566 | 2864873 |
|  |  | 1889740 |
|  |  | 1027699 |
| *ATM* | 1800889 | 664143 |
|  | 1801516 | 189037 |
|  | 170548 | 664677 |
|  | 4585 | 3092993 |
|  |  | 228589 |
| *AXIN2* | 2240308 |  |
|  | 11868547 |  |
|  | 3923087 |  |
|  | 4541111 |  |
|  | 7210356 |  |
|  | 11867417 |  |
|  | 4128941 |  |
| *BAK1* | 210135 | 513349 |
|  | 210145 |  |
| *BARD1* | BARD1-02 | 5031011 |
|  | 2070094 |  |
|  | 2229571 |  |
|  | 2070096 |  |
| *BAX* | 4645887 |  |
|  | 905238 |  |
| *BCL2L1* | 3181073 | 1994251 |
|  | 1484994 |  |
| *BCL6* | 3172469 |  |
|  | 1464645 |  |
|  | 1474326 |  |
|  | 3774309 |  |
|  | 3774306 |  |
| *BCR* | 12233352 |  |
|  | 140504 |  |
| *BHMT* | 585800 |  |
|  | 567754 |  |
|  | 617219 |  |
| *BIC* | 928883 | 915860 |
|  | 767649 | 1893650 |
|  | 12482371 | 2829801 |
|  | BIC-21 | 2829803 |
|  | 4143370 |  |
|  | 4817027 |  |
| *BIRC3* | 3758841 |  |
|  | 3460 |  |
| *BLM* | 2238335 | 2072352 |
|  | 2270132 |  |
|  | 2073919 |  |
|  | 389480 |  |
|  | 2072351 |  |
|  | 16944831 |  |
| *BRCA1* | 1799950 | 16940 |
|  | 4986852 | 1060915 |
|  | 799923 | 1799949 |
|  | 8176212 | 1799966 |
| *BRCA2* | 144848 | 15869 |
|  | 1801406 |  |
|  | 543304 |  |
|  | 1799955 |  |
|  | 1799943 |  |
|  | 206147 |  |
|  | 1207953 |  |
| *BRIP1* | 2048718 | 4986763 |
|  | 4986764 |  |
|  | 4986765 |  |
|  | 1015771 |  |
|  | 4988340 |  |
| *BZRP* | 6971 | 113515 |
|  | 3937387 |  |
| *CALCR* | 1801197 |  |
|  | 2074122 |  |
| *CARD15* | 2066850 | 748855 |
|  | 2066843 |  |
|  | 1077861 |  |
|  | 2067085 |  |
| *CASP3* | 3087455 |  |
|  | 1405938 |  |
|  | 1049216 |  |
|  | 6948 |  |
| *CASP8* | 2349070 |  |
|  | 2293554 |  |
|  | 1035142 |  |
| *CASP9* | 1052576 |  |
|  | 2020902 |  |
|  | 2020898 |  |
| *CASR* | 1042636 |  |
|  | 1965357 |  |
|  | 2270916 |  |
|  | 2279802 |  |
|  | 2270917 |  |
|  | 4678045 |  |
|  | 3749208 |  |
| *CAT* | 769214 | 769218 |
|  | 769217 | 1049982 |
|  | 475043 |  |
|  | 9282626 |  |
| *CAV1* | 2215448 | 6950798 |
|  | 8713 |  |
|  | 1049334 |  |
|  | 1049337 |  |
|  | 10257125 |  |
|  | cav1_23 |  |
| *CBR1* | 25678 |  |
|  | 1005695 |  |
|  | 2156406 |  |
| *CBS* | 234706 |  |
|  | 12613 |  |
|  | 6586282 |  |
| *CCL5* | 2107538 |  |
|  | 2280789 |  |
| *CCNA2* | 769242 |  |
|  | 1396080 |  |
|  | 3217773 |  |
| *CCND1* | 678653 |  |
|  | 603965 |  |
|  | 7177 |  |
| *CCND3* | 9529 |  |
|  | 2479717 |  |
| *CCNH* | 2266690 |  |
|  | 3093816 |  |
| *CCR2* | 1799864 | 3138042 |
|  | 1799865 | 1800024 |
| *CCR3* | 4987053 |  |
|  | 3091312 |  |
| *CCR5* | 2734648 |  |
|  | 1799987 |  |
| *CD40* | 1535045 |  |
|  | 3765459 |  |
| *CD80* | 2228017 |  |
|  | 1385520 |  |
|  | 9282638 |  |
| *CD81* | 708155 |  |
|  | 810225 |  |
| *CD86* | 1129055 |  |
|  | 9282641 |  |
| *CDH1* | 9282650 |  |
|  | 1801026 |  |
| *CDK5* | 2069456 | 10245199 |
|  | 1549760 |  |
| *CDKN2A* | 3088440 | 3731198 |
|  | 2518719 | 3731211 |
|  | 3731246 | 3731217 |
|  | 3731239 |  |
|  | 2518720 |  |
|  | 2811708 |  |
|  | 3218020 |  |
| *CETP* | 820299 |  |
|  | 1801706 |  |
|  | 289717 |  |
| *CFH* | 800292 |  |
|  | 2274700 |  |
|  | 1065489 |  |
|  | 1329423 |  |
|  | 2300430 |  |
| *CGA* | 6631 |  |
|  | 4986869 |  |
|  | 6155 |  |
|  | 932742 |  |
| *CHEK1* | 558351 |  |
|  | 506504 |  |
|  | 491528 |  |
| *COL18A1* | 7499 |  |
|  | 2236451 |  |
|  | 2236467 |  |
| *COMT* | 4680 | 6269 |
|  | 4646312 | 4646310 |
|  | 7290221 |  |
|  | 2240716 |  |
|  | 6518591 |  |
|  | 9306230 |  |
| *CRP* | 1800947 |  |
|  | 1205 |  |
| *CSF1R* | 2228422 |  |
|  | 3829987 |  |
|  | 10079250 |  |
| *CSF3* | 1042658 |  |
|  | 2227338 |  |
| *CSTF1* | 6064389 | 6099129 |
|  | 16979877 | 6064387 |
| *CTH* | 663465 | 663649 |
|  | 6413471 |  |
|  | 473334 |  |
|  | 515064 |  |
|  | 559062 |  |
| *CTLA4* | 231775 |  |
|  | 3087243 |  |
|  | 11571317 |  |
|  | 11571315 |  |
|  | 11571316 |  |
|  | 4553808 |  |
|  | 5742909 |  |
| *CTNNB1* | 11564437 | 11564452 |
|  | 1798794 | 11564465 |
|  | 4135385 | 2371452 |
|  | 9813198 | 2953 |
|  | 9883073 | 3864004 |
|  |  | 4533622 |
|  |  | 5743395 |
|  |  | 11129895 |
|  |  | 1880481 |
| *CX3CR1* | 3732379 |  |
|  | 3732378 |  |
| *CYP1A1* | 2606345 |  |
|  | 4646421 |  |
|  | 2198843 |  |
|  | 2472299 |  |
|  | 17861115 |  |
| *CYP17A1* | 743572 |  |
|  | 10883782 |  |
|  | 284849 |  |
|  | 4919682 |  |
|  | 4919687 |  |
|  | 619824 |  |
| *CYP19A1* | 700518 | 1065779 |
|  | 2304463 | 10046 |
|  | 4646 | 767199 |
|  | 730154 | 1004984 |
|  | 1004983 | 2414096 |
|  | 12907866 | 2445765 |
|  | 28566535 |  |
|  | 2446405 |  |
|  | 2470144 |  |
|  | 6493494 |  |
|  | 727479 |  |
|  | 749292 |  |
| *CYP24A1* | 2248359 |  |
|  | 2259735 |  |
|  | 2296241 |  |
|  | 751087 |  |
| *CYP1B1* | 1800440 | 162562 |
|  | 10916 |  |
|  | 10175368 |  |
|  | 162556 |  |
|  | 162555 |  |
|  | 163077 |  |
|  | 163086 |  |
|  | 1367696 |  |
| *CYP7B1* | 3779870 |  |
|  | 1376772 |  |
|  | 1451868 |  |
|  | CYP7B1-06 |  |
| *CYP2E1* | 2070676 |  |
|  | 8192766 |  |
| *DHDH* | 4987162 |  |
|  | 2270939 |  |
| *DHFR* | DHFR-07 |  |
|  | 865646 |  |
|  | 1650697 |  |
| *DIO1* | 1883454 |  |
|  | 2235544 |  |
| *DRD2* | 1799978 |  |
|  | 1079597 |  |
| *DRD4* | 916457 |  |
|  | 4987059 |  |
| *EDN1* | 5369 |  |
|  | 5370 |  |
| *EFNB3* | 3744263 |  |
|  | 3744262 |  |
| *EGF* | 2237051 |  |
|  | 971696 |  |
|  | 4444903 |  |
| *EGFR* | 1140475 |  |
|  | 2293347 |  |
|  | 2017000 |  |
| *EPHX1* | 2234922 |  |
|  | 1051740 |  |
|  | 2260863 |  |
|  | 1051741 |  |
|  | 2671272 |  |
|  | 2854461 |  |
|  | 2854456 |  |
|  | 3738043 |  |
| *ERCC1* | 11615 |  |
|  | 3212948 |  |
| *ERCC2* | 28365048 |  |
|  | 1799787 |  |
|  | 6966 |  |
| *ERCC3* | 4150416 |  |
|  | 4150474 |  |
| *ERCC4* | 1800067 |  |
|  | 1799800 |  |
| *ERCC5* | 1047768 |  |
|  | 17655 |  |
|  | 2227869 |  |
| *ESR1* | 2077647 |  |
|  | 2228480 |  |
|  | 1801132 |  |
|  | 3798577 |  |
|  | 2071454 |  |
|  | 2273206 |  |
|  | 3798758 |  |
|  | 488133 |  |
|  | 9340770 |  |
| *ESR2* | 4986938 |  |
|  | 3020450 |  |
| *EXO1* | 4149963 |  |
|  | 735943 |  |
| *FANCA* | 2239359 | 1061646 |
|  | 12931267 | 2239360 |
|  | 2159116 | 2016571 |
|  | 3785275 | 886951 |
|  | 7195906 | 17227099 |
|  |  | 7203907 |
| *FAS* | 1324551 |  |
|  | 1468063 |  |
|  | 2234768 |  |
| *FBXW7* | 2714805 | 2676330 |
|  | 2714804 | 2292743 |
|  | 2676329 |  |
| *FOS* | 7101 |  |
|  | 1063169 |  |
|  | 4645856 |  |
| *FOXC1* | 2235718 |  |
|  | 984253 |  |
|  | 2745599 |  |
|  | 9405496 |  |
|  | 2235716 |  |
|  | 6928414 |  |
| *FZD7* | 13034206 | FZD7-06 |
|  | FZD7-15 | 12474408 |
|  | 1207955 |  |
|  | 4673222 |  |
| *GATA3* | 2229359 | 569421 |
|  | 570730 | 1149901 |
|  | 520236 |  |
|  | 422628 |  |
|  | 406103 |  |
|  | 528778 |  |
|  | 10905277 |  |
|  | 10752126 |  |
|  | 3781093 |  |
|  | 1269486 |  |
| *GDF15* | 1059519 |  |
|  | 1059369 |  |
| *GGH* | 719235 |  |
|  | 1031552 |  |
| *GHR* | 6179 | 2940944 |
|  | 6180 | 2972780 |
|  | 4451056 | 6873545 |
|  | GHR-113 | 6897530 |
|  | 7732059 | 6878512 |
|  | 28943882 | 1858136 |
|  | 2940913 |  |
|  | 2940930 |  |
|  | 2972392 |  |
|  | 2972395 |  |
|  | 2972418 |  |
|  | 7712701 |  |
|  | 7735889 |  |
|  | 2972419 |  |
|  | 28943889 |  |
|  | 7579 |  |
|  | 6413428 |  |
| *GPX2* | 6784820 | 6997 |
|  | 3448 | 17880380 |
|  | 1800668 | 10133054 |
|  | 1800669 | 10133290 |
|  | GPX2-07 | 2071566 |
|  | 12172810 | 4902345 |
|  | 2296327 | 8897 |
|  | 2737844 |  |
| *GPX3* | 1946234 | 8177404 |
|  | 8177447 | 2277940 |
|  | 869975 |  |
|  | 2042235 |  |
|  | 8177426 |  |
| *GPX4* | 3746165 | 757228 |
|  | 4807542 |  |
|  | 8178977 |  |
| *GSK3B* | 1719888 | 1154597 |
|  | 334555 | 12630592 |
|  | 334559 | 1381841 |
|  | 4624596 | 1719889 |
|  | 17810235 | 1719895 |
|  | 17810302 | 1732170 |
|  | 9873477 | 2873950 |
|  | 3755557 | 4688046 |
|  |  | 6779828 |
|  |  | 7617372 |
|  |  | 9878473 |
|  |  | 6438553 |
|  |  | 6781942 |
|  |  | 10934500 |
|  |  | 10934503 |
|  |  | 1574154 |
|  |  | 16830683 |
|  |  | 16830689 |
|  |  | 17204605 |
|  |  | 17204878 |
|  |  | 17810676 |
|  |  | 1870931 |
|  |  | 2319398 |
|  |  | 334535 |
|  |  | 3732361 |
|  |  | 4072520 |
|  |  | 4688047 |
|  |  | 6770314 |
|  |  | 7620750 |
|  |  | 9851174 |
| *GSTA4* | 405729 |  |
|  | 367836 |  |
|  | 4986947 |  |
|  | 543613 |  |
| *GSTM3* | 7483 |  |
|  | 2234696 |  |
|  | 1537234 |  |
| *GSTP1* | 947894 |  |
|  | 1799811 |  |
| *GSTZ1* | GSTZ1-02 |  |
|  | 1046428 |  |
| *HFE* | 1799945 |  |
|  | 1572982 |  |
|  | 707889 |  |
| *HSD3B1* | 10754400 | 1998182 |
|  | 6428830 | 2064902 |
|  | 6667572 | 4659182 |
| *HSD17B1* | 597255 | 598126 |
|  | 676387 |  |
| *HSD17B2* | 1424151 |  |
|  | 723012 |  |
| *HSD17B4* | 28943585 | 17145464 |
|  | 28943596 | 246965 |
|  | 2546210 |  |
|  | 2451818 |  |
|  | 32659 |  |
|  | 3797372 |  |
|  | 384346 |  |
|  | 7737181 |  |
| *HSD3B2* | 1361530 |  |
|  | 12411115 |  |
|  | 4659175 |  |
|  | 879332 |  |
|  | 1417608 |  |
|  | 1417604 |  |
| *HTR1B* | 6296 |  |
|  | 130058 |  |
| *HTR1D* | 605367 |  |
|  | 676643 |  |
|  | 6300 |  |
| *ICAM1* | 5498 |  |
|  | 281432 |  |
|  | 3093032 |  |
|  | 5030390 |  |
| *IFNAR2* | 3153 | 2236757 |
|  | 7279064 |  |
| *IFNGR1* | 11914 |  |
|  | 3799488 |  |
| *IGF1* | 2162679 | 2373721 |
|  | 5742629 | 978458 |
|  | 4764883 | 5742667 |
|  | 5742694 |  |
|  | 5742714 |  |
|  | 5742665 |  |
| *IGF1R* | 2229765 | 2175795 |
|  | 2137680 |  |
|  | 907806 |  |
|  | 9282715 |  |
|  | 3743259 |  |
|  | 3743260 |  |
| *IGF2* | 3213216 | 734351 |
|  | 2230949 |  |
|  | 3213221 |  |
|  | 3213223 |  |
| *IGF2AS* | 1003483 |  |
|  | 3741211 |  |
|  | 3741212 |  |
| *IGF2R* | 998075 | 998074 |
|  | 1803989 |  |
|  | 629849 |  |
|  | 1570070 |  |
|  | 2282140 |  |
| *IGFBP2* | 2270360 |  |
|  | 1106037 |  |
|  | 2372848 |  |
| *IGFBP5* | 2241193 |  |
|  | 1978346 |  |
| *IGFBP6* | 12821902 | 7974876 |
|  | 822688 |  |
| *IL1A* | 17561 |  |
|  | 2071374 |  |
| *IL1B* | 1143627 | 1143634 |
|  | 1071676 |  |
|  | 3136558 |  |
| *IL1RN* | 454078 | 419598 |
|  | 380092 |  |
| *IL2* | 2069762 |  |
|  | 2069763 |  |
| *IL4* | 2243248 | 2243250 |
|  | 2070874 | 2243290 |
|  |  | 2243268 |
| *IL4R* | 1805011 |  |
|  | 1805012 |  |
|  | 1805015 |  |
|  | 1805016 |  |
|  | 8832 |  |
|  | 2057768 |  |
|  | 3024544 |  |
| *IL7R* | 1494555 |  |
|  | 7737000 |  |
| *IL8* | 4073 |  |
|  | 2227306 |  |
|  | 2227549 |  |
| *IL10* | 1800871 | 1800896 |
|  | 3024509 | 3024491 |
|  | 3024496 |  |
|  | 3021094 |  |
|  | 1800890 |  |
| *IL10RA* | 9610 |  |
|  | 2229114 |  |
| *IL12B* | 3212227 |  |
|  | 730690 |  |
| *IL13* | 20541 |  |
|  | 1881457 |  |
|  | 1800925 |  |
|  | 1295686 |  |
| *IL15* | 2254514 | 1057972 |
|  | 10833 |  |
|  | 1493013 |  |
|  | 2857261 |  |
| *IL15RA* | 2296135 |  |
|  | 2228059 |  |
|  | 2296141 |  |
|  | 3136614 |  |
| *INSR* | 1799817 | 1035942 |
|  | 891087 |  |
|  | 1051690 |  |
|  | 2860175 |  |
|  | 919275 |  |
|  | 3815901 |  |
|  | 3745551 |  |
|  | 8110533 |  |
|  | 1035940 |  |
|  | INSR-59 |  |
|  | 3745545 |  |
| *IRF3* | 2304204 | 2304206 |
|  | 7251 |  |
| *IRS1* | 1801278 |  |
|  | 1366757 |  |
|  | 9282766 |  |
| *JAK3* | 3008 |  |
|  | 3212711 |  |
|  | 3212752 |  |
| *KRAS* | 4623993 | 10842515 |
|  | 10842518 | 2970532 |
|  | 11047902 | 10505980 |
|  | 1137196 | 17473423 |
|  | 17388148 | 13096 |
|  | 4368021 | 4246229 |
|  | 7133640 | 712 |
|  | 7973746 | 11047918 |
|  |  | 12226937 |
|  |  | 12228277 |
|  |  | 17329025 |
|  |  | 17329424 |
|  |  | 6487461 |
|  |  | 9266 |
| *LCAT* | 5923 |  |
|  | 1109166 |  |
| *LDLR* | 1003723 |  |
|  | 14158 |  |
|  | 2116898 |  |
|  | 5930 |  |
|  | 5925 |  |
| *LEPR* | 1137100 |  |
|  | 7602 |  |
|  | 1137101 |  |
|  | 1887285 |  |
| *LIG1* | 20580 |  |
|  | 13436 |  |
|  | 20579 |  |
|  | 3729512 |  |
|  | 156641 |  |
| *LIPC* | 1800588 | 1077834 |
|  | 3825776 |  |
|  | 1968687 |  |
|  | 6083 |  |
|  | 2242064 |  |
|  | 6074 |  |
|  | 2242066 |  |
|  | 1869145 |  |
|  | 1968689 |  |
| *LITAF* | 7102 |  |
|  | 4280262 |  |
| *LMO2* | 3740617 |  |
|  | 3781577 |  |
|  | 3740616 |  |
| *LPL* | 263 | 328 |
|  | 326 |  |
|  | 316 |  |
|  | 1059507 |  |
|  | 325 |  |
|  | 327 |  |
| *LRP5* | 312016 | 608343 |
|  | 491347 |  |
|  | 607887 |  |
|  | 3736228 |  |
| *LRP6* | 3782528 |  |
|  | 2075241 |  |
| *LTA* | 909253 |  |
|  | 3093546 |  |
| *MASP1* | 3774268 | 12635264 |
|  | 3733001 | 13089330 |
|  | 1001073 | 3864099 |
|  | 13094773 | 4376034 |
|  | 1533593 | 698105 |
|  | 3105782 | 7609662 |
|  | 696405 |  |
|  | 698079 |  |
|  | 698090 |  |
|  | 710459 |  |
| *MBD2* | 7614 | 603097 |
|  | 1145315 |  |
|  | 609791 |  |
| *MBL2* | 5030737 | 11003125 |
|  | 1838066 | 2099902 |
|  | 930508 | 12264958 |
|  | 7096206 |  |
|  | 10082466 |  |
|  | 1031101 |  |
|  | 11003124 |  |
|  | 10824793 |  |
| *MET* | 41736 |  |
|  | 13223756 |  |
|  | 11762213 |  |
|  | MET-16 |  |
| *MGMT* | 2308327 |  |
|  | 12917 |  |
|  | MGMT-12 |  |
|  | 2296675 |  |
| *MLH1* | 1799977 |  |
|  | 2286940 |  |
| *MMP1* | 5854 | 10488 |
|  | 5031036 |  |
|  | 2071230 |  |
| *MSH2* | 17036577 | 2303428 |
|  | 1863332 | 1981928 |
|  | 2042649 | 3771281 |
|  | 3821227 | 4608577 |
|  | 4952887 | 7585925 |
|  | 6544991 | 7602094 |
|  | 7607076 | 17036614 |
| *MSH3* | 1805355 | 836802 |
|  | 3797896 |  |
|  | 26279 |  |
|  | 32983 |  |
|  | 1677649 |  |
| *MSH6* | 3136228 |  |
|  | 1800935 |  |
| *MSR1* | 414580 |  |
|  | 971594 |  |
| *MTHFR* | 1801133 |  |
|  | 2066470 |  |
|  | 12121543 |  |
| *MTR* | 1805087 |  |
|  | 2275565 |  |
|  | 2275566 |  |
| *MTRR* | 9332 | 2287780 |
|  | 10380 |  |
|  | 1802059 |  |
|  | 8659 |  |
|  | 2287779 |  |
| *MX1* | 2280807 | 1050008 |
|  | 458582 | 469390 |
|  | 469270 | 2072683 |
|  | 469304 | 455599 |
|  | 2070229 |  |
| *MYBL2* | MYBL2-03 | 619289 |
|  | 419842 | 420755 |
|  | 285171 |  |
|  | 385345 |  |
|  | 826950 |  |
|  | 285164 |  |
| *MYO5A* | 1058219 |  |
|  | 2290336 |  |
|  | 2242058 |  |
| *NBS1* | 1805794 | 1063045 |
|  | 1063053 |  |
|  | 867185 |  |
| *NCF2* | 2274064 | 2296164 |
|  | 699244 |  |
| *NCOA3* | 396221 |  |
|  | 427967 |  |
|  | 2076546 |  |
| *NFKB1* | 3774932 | 230532 |
|  | 3774937 |  |
|  | 230496 |  |
|  | 230547 |  |
|  | 4648059 |  |
| *NFKBIE* | 730775 | 483536 |
|  | 2282151 |  |
|  | 513688 |  |
| *NINJ1* | 1127851 |  |
|  | 1127857 |  |
| *NOS2A* | 2297518 |  |
|  | 9282801 |  |
| *NOS3* | 1799983 |  |
|  | 3918226 |  |
| *NQO1* | 689453 | 689452 |
|  | 10517 |  |
| *NR1H4* | 35724 |  |
|  | NR1H4-18 |  |
| *OCA2* | 1800404 |  |
|  | 1800407 |  |
|  | 1900758 |  |
| *OGG1* | 125701 |  |
|  | 2304277 |  |
| *OPRD1* | 760589 |  |
|  | 204076 |  |
| *OPRM1* | 1799971 |  |
|  | 607759 |  |
|  | 562859 |  |
|  | 9282821 |  |
| *PAK6* | 2242119 | 748556 |
|  | 2242120 |  |
|  | 936216 |  |
|  | 11636097 |  |
|  | 900055 |  |
| *PARP1* | 1805415 | 1136410 |
|  | 747657 | 1805407 |
|  | 747659 | 1805414 |
| *PARP4* | 13428 |  |
|  | 6413414 |  |
|  | 1539096 |  |
|  | 750771 |  |
|  | 1807111 |  |
| *PCNA* | 25406 | 17352 |
|  | 17349 |  |
| *PCTP* | 2114443 |  |
|  | 12948867 |  |
| *PGR* | 613120 | 1042839 |
|  | 9282823 | 1042838 |
|  | 492457 | 565186 |
|  | 516693 |  |
|  | 529359 |  |
|  | 543215 |  |
|  | 572483 |  |
|  | 1870019 |  |
|  | 474320 |  |
|  | 481775 |  |
|  | 561650 |  |
|  | 568157 |  |
|  | 660541 |  |
|  | 10895068 |  |
| *PIM1* | 10507 | 1757000 |
|  | 262933 |  |
|  | 12197850 |  |
| *PIN1* | 2233678 | 2233679 |
|  | 1985604 |  |
|  | 2010457 |  |
|  | 889162 |  |
| *PLA2G6* | 4376 | 132987 |
|  | 2016755 |  |
|  | 84473 |  |
| *PMS1* | 5743030 | 1233302 |
|  | 1233258 | 1233297 |
|  | 1233288 | 256564 |
|  | 1233255 | 1233284 |
|  | 12618262 | 1233299 |
|  | 256567 | 256550 |
|  | 5742926 | 256552 |
|  | 5743072 | 256563 |
|  |  | 5742938 |
|  |  | 5743112 |
|  |  | 5743116 |
|  |  | 1233291 |
| *PMS2* | 3735295 |  |
|  | 2345060 |  |
|  | 6463524 |  |
| *POLB* | 3136717 | 2979895 |
|  | 2953983 |  |
| *POT1* | POT1-03 | 727506 |
|  | 10244817 | 6959712 |
|  | 6466966 | 7784168 |
|  |  | 10263573 |
|  |  | 10250202 |
|  |  | 1034794 |
|  |  | POT1-37 |
| *PPARG* | 2938392 |  |
|  | 1175541 |  |
|  | 1801282 |  |
| *PTEN* | 701848 |  |
|  | 1903858 |  |
| *PTGS2* | 689466 |  |
|  | 20417 |  |
|  | 5277 |  |
|  | 5275 |  |
|  | 4648276 |  |
| *PTH* | 6256 |  |
|  | 177706 |  |
|  | 6254 |  |
| *RAB15* | 2277502 |  |
|  | 3742599 |  |
|  | 3825644 |  |
| *RAD23B* | 1805335 |  |
|  | 1805330 |  |
|  | 1805329 |  |
|  | 1805334 |  |
| *RAD51* | 1801320 | 2304579 |
|  | 4924496 | 2412546 |
|  | 2619679 | 11852786 |
|  | 2619681 | 2412547 |
|  |  | 4144242 |
| *RAD52* | 11226 |  |
|  | 6413436 |  |
| *RB1CC1* | 17845549 | 2305427 |
|  | 17337252 |  |
|  | RB1CC1-50 |  |
| *RERG* | RERG-03 | 6488766 |
|  | 17834986 | 1045733 |
|  | RERG-29 |  |
|  | 10160846 |  |
|  | 2216225 |  |
|  | 3748302 |  |
|  | 715398 |  |
|  | 1055151 |  |
|  | 2193174 |  |
|  | 767201 |  |
| *RET* | 1800858 |  |
|  | 1800860 |  |
| *RGS6* | 2238284 |  |
|  | 3784058 |  |
|  | 2238280 |  |
| *RGS17* | 2295231 |  |
|  | 3870366 |  |
| *RNASEL* | 11072 |  |
|  | 486907 |  |
| *ROS1* | 2243 | 581235 |
|  | 2243377 | 574664 |
|  | 1998206 | 497186 |
|  | 498251 |  |
| *RXRA* | 1536475 |  |
|  | 1805352 |  |
| *RXRB* | 2076310 |  |
|  | 2072915 |  |
| *SAT2* | 13894 |  |
|  | 858520 |  |
| *SCARB1* | 3924313 |  |
|  | 4765181 |  |
|  | 4765621 |  |
|  | 865716 |  |
|  | 989892 |  |
| *SCUBE2* | 3751052 |  |
|  | 2003906 |  |
|  | 3751058 |  |
| *SFTPD* | 721917 |  |
|  | 2243639 |  |
| *SHBG* | 6259 |  |
|  | 6257 |  |
|  | 727428 |  |
|  | 858517 |  |
| *SLAMF1* | 2295612 |  |
|  | 164283 |  |
|  | 1061217 |  |
| *SLC4A2* | 6464120 |  |
|  | 13240966 |  |
| *SLC6A3* | 6413429 |  |
|  | 2652511 |  |
|  | 6347 |  |
|  | 460700 |  |
| *SLC19A1* | 1051266 |  |
|  | 1051298 |  |
| *SLC23A1* | SLC23A1-05 | 4257763 |
|  | 10063949 |  |
|  | 6596471 |  |
|  | 4315920 |  |
|  | 11738738 |  |
| *SLC23A2* | 1110277 | 6084957 |
|  | SLC23A2-02 |  |
|  | 1776964 |  |
|  | 4987219 |  |
|  | 1715364 |  |
|  | 12479919 |  |
|  | 4813725 |  |
| *SLC39A2* | 2234636 | 2149666 |
|  | 945352 |  |
| *SOAT2* | 2280699 | 17123210 |
|  | 2280698 |  |
| *SOD2* | 1799725 |  |
|  | 5746081 |  |
| *SRA1* | 801459 |  |
|  | 801460 |  |
|  | 10463297 |  |
| *STK6 (AURKA)* | 1047972 | 2298016 |
|  | 2273535 |  |
|  | 6024840 |  |
|  | AURKA-08 |  |
|  | 8173 |  |
|  | 10485805 |  |
| *TEP1* | 1760898 |  |
|  | 1760904 |  |
|  | 1713449 |  |
|  | 1760897 |  |
|  | 872072 |  |
|  | 872074 |  |
| *TERF1* | 2306492 | 2306494 |
|  | TERF1-02 |  |
|  | 3863242 |  |
|  | 10106086 |  |
| *TERF2* | 153045 |  |
|  | TERF2-03 |  |
|  | 251796 |  |
| *TERT* | 2075786 |  |
|  | 2853690 |  |
|  | 2735940 |  |
|  | 2853677 |  |
|  | 13167280 |  |
|  | 1801075 |  |
|  | 3816659 |  |
| *TGFB1* | 1800471 |  |
|  | 2241719 |  |
|  | 1800469 |  |
| *TGFBR1* | 928180 | 868 |
|  | 334358 |  |
| *TGM1* | 2229463 |  |
|  | 2855006 |  |
| *TLR2* | 3804099 |  |
|  | 3804100 |  |
|  | 4696480 |  |
| *TNF* | 1800629 |  |
|  | 1800630 |  |
|  | 1799964 |  |
|  | 3093661 |  |
| *TNFRSF10A* | 2235126 |  |
|  | 4871857 |  |
| *TNKS* | TNKS-03 | TNKS-01 |
|  | TNKS-05 | 12542457 |
|  | 6992432 | TNKS-124 |
|  | 6985140 | 6601361 |
|  | 7006985 |  |
|  | 7462910 |  |
|  | 7001395 |  |
|  | 9644708 |  |
|  | TNKS-26 |  |
|  | 7462102 |  |
|  | 7464476 |  |
|  | TNKS-36 |  |
|  | 11249938 |  |
|  | TNKS-46 |  |
|  | TNKS-64 |  |
|  | 13276464 |  |
| *TP53* | 8079544 | 2287498 |
|  | 12951053 |  |
|  | 1614984 |  |
|  | 2909430 |  |
|  | 2078486 |  |
|  | 17886268 |  |
|  | 17885803 |  |
|  | 2287499 |  |
|  | 1641512 |  |
|  | 1624085 |  |
|  | 1641535 |  |
| *TP53I3* | 2303287 | 10170774 |
|  | 4149372 |  |
|  | 7603220 |  |
|  | 4149371 |  |
| *TP73L* | 17514215 |  |
|  | 9840360 |  |
|  | 6789961 |  |
|  | 6790167 |  |
|  | 7653848 |  |
|  | 1345186 |  |
|  | 7613791 |  |
|  | TP73L-46 |  |
|  | TP73L-47 |  |
|  | TP73L-52 |  |
| *TSG101* | 12574333 | 2292179 |
|  | 2279902 | 2279900 |
|  | 2291752 |  |
|  | 2292176 |  |
| *TYMS* | 2790 | 699517 |
|  | 1059394 |  |
| *UCP3* | 2075577 |  |
|  | 1800849 |  |
| *VCAM1* | 1041163 | 2392221 |
|  | 3176879 |  |
| *VDR* | 2239185 |  |
|  | 757343 |  |
| *VEGF* | 3025039 |  |
|  | 25648 |  |
|  | 1005230 |  |
| *VIL2* | 901369 |  |
|  | 3123109 |  |
| *WRN* | 2230009 | 1801195 |
|  | 1346044 |  |
|  | 2725349 |  |
|  | 1800392 |  |
| *XBP1* | 2097461 | 2239815 |
|  | 2267131 | 3788409 |
| *XPC* | 2228001 |  |
|  | 2228000 |  |
|  | 3731151 |  |
| *XRCC3* | 1799796 |  |
|  | 1799794 |  |
| *XRCC4* | 3777015 | 1805377 |
|  | 2075685 |  |
|  | 2662238 |  |
|  | 2891980 |  |
| *XRCC5* | 1051685 |  |
|  | 2440 |  |
|  | 828910 |  |
|  | 828702 |  |
|  | 207916 |  |
|  |  |  |

* If an rs number has not yet been assigned, an internal SNP ID number E number from the SNP500Cancer project ([http://snp500cancer.nci.nih.gov](http://snp500cancer.nci.nih.gov/)) was used.

** SNPs excluded because of pairwise correlation coefficients >0.90 with other SNPs included in the models.
