# Supplementary material for: Large-Scale Evaluation of Candidate Genes Identifies Associations between VEGF Polymorphisms and Bladder Cancer Risk
Source: PLoS Genet. 2007 Feb 23;3(2):e29. doi: 10.1371/journal.pgen.0030029 (PMC1802828; doi:10.1371/journal.pgen.0030029)
Supplement: Table S2 — (34 KB DOC). [file pgen.0030029.st002.doc]

Supplementary Table 2: SNP-based analyses: association between bladder cancer risk and SNPs with a p-value from a trend test or a 2-df LRT ≤ 0.01 in the Spanish Bladder Cancer Study

MAF: minor allele frequency among the control population.

*The common homozygote genotype is the reference category. ORs are adjusted for age, region, gender and smoking status.

*VEGF:* Vascular endothelial growth factor; *GHR:* Growth hormone receptor; *PTH:* Parathyroid hormone receptor 1 *ROS1:* v-ros UR2 sarcoma virus oncogene homolog 1 (avian); *PLA2G6:* phospholipase A2, group VI; *XRCC1*: X-ray repair complementing defective repair in Chinese hamster cells 1; *ZNF350:* Zinc finger protein 350; *PLA2G6*: phospholipase A2, group VI (cytosolic, calcium-independent); *RB1CC1:* RB1-inducible coiled-coil 1; *TERT*: telomerase reverse transcriptase; *XRCC4*: X-ray repair complementing defective repair in Chinese hamster cells 4; *FZD7*: frizzled homolog 7 (Drosophila); *CETP*: cholesteryl ester transfer protein, plasma ; *CYP24A1*: cytochrome P450, family 24, subfamily A, polypeptide 1; *LIPC*: lipase, hepatic; *ESR1*: estrogen receptor 1; *STK11:* Serine/threonine kinase 11; *ARHGDIB:* Rho GDP dissociation inhibitor (GDI) beta; *HSD17B4*: hydroxysteroid (17-beta) dehydrogenase 4; *GPX4*: glutathione peroxidase 4 (phospholipid hydroperoxidase); *STAT1:* Signal transducer and activator of transcription 1, 91kDa; *SHBG*: sex hormone-binding globulin; *CYP7B1*: cytochrome P450, family 7, subfamily B, polypeptide 1.
