# Supplementary material for: Large-Scale Evaluation of Candidate Genes Identifies Associations between VEGF Polymorphisms and Bladder Cancer Risk
Source: PLoS Genet. 2007 Feb 23;3(2):e29. doi: 10.1371/journal.pgen.0030029 (PMC1802828; doi:10.1371/journal.pgen.0030029)
Supplement: Table S3 — (233 KB DOC). [file pgen.0030029.st003.doc]

Supplementary Table 3: Association between common variants in VEGF and bladder cancer risk in the Spanish Bladder Cancer Study.

| rs number nucleotide change | Genotype | Cases | % | Controls | % | OR | 95% CI | | P | P trend |
| --- | --- | --- | --- | --- | --- | --- | --- | --- | --- | --- |
| rs9394963 | GG | 404 | 46% | 391 | 45% | 1.00 |  |  |  |  |
| -19592G>T | GT | 398 | 45% | 375 | 43% | 1.02 | 0.83 | 1.25 | 0.866 |  |
|  | TT | 85 | 10% | 104 | 12% | 0.85 | 0.61 | 1.19 | 0.357 | 0.551 |
| rs833052 | CC | 677 | 75% | 687 | 78% | 1.00 |  |  |  |  |
| -15648A>C | CA | 209 | 23% | 189 | 21% | 1.13 | 0.89 | 1.43 | 0.314 |  |
|  | AA | 19 | 2% | 8 | 1% | 2.52 | 1.06 | 5.97 | 0.036 | 0.062 |
| rs866236 | CC | 503 | 57% | 488 | 56% | 1.00 |  |  |  |  |
| -12027T>C | CT | 315 | 36% | 329 | 38% | 0.94 | 0.76 | 1.16 | 0.567 |  |
|  | TT | 63 | 7% | 54 | 6% | 1.08 | 0.72 | 1.61 | 0.706 | 0.916 |
| rs833057 | TT | 522 | 59% | 509 | 58% | 1.00 |  |  |  |  |
| -11953G>T | TG | 310 | 35% | 314 | 36% | 0.96 | 0.78 | 1.18 | 0.711 |  |
|  | GG | 53 | 6% | 51 | 6% | 1.01 | 0.66 | 1.54 | 0.965 | 0.831 |
| rs1109324 | GG | 623 | 70% | 645 | 74% | 1.00 |  |  |  |  |
| -9228G>T | GT | 243 | 27% | 221 | 25% | 1.12 | 0.90 | 1.41 | 0.305 |  |
|  | TT | 25 | 3% | 10 | 1% | 2.74 | 1.26 | 5.98 | 0.011 | 0.034 |
| rs1547651 | AA | 619 | 69% | 638 | 73% | 1.00 |  |  |  |  |
| -8339A>T | AT | 251 | 28% | 231 | 26% | 1.12 | 0.90 | 1.40 | 0.313 |  |
|  | TT | 27 | 3% | 9 | 1% | 3.01 | 1.36 | 6.63 | 0.006 | 0.027 |
| rs833060 | GG | 457 | 52% | 461 | 53% | 1.00 |  |  |  |  |
| -3834G>T | GT | 367 | 41% | 353 | 41% | 1.00 | 0.82 | 1.23 | 0.962 |  |
|  | TT | 62 | 7% | 57 | 7% | 1.10 | 0.74 | 1.64 | 0.641 | 0.740 |
| rs699947 | CC | 261 | 27% | 268 | 29% | 1.00 |  |  |  |  |
| -2594A>C | CA | 471 | 49% | 447 | 48% | 1.12 | 0.90 | 1.41 | 0.306 |  |
|  | AA | 220 | 23% | 214 | 23% | 1.13 | 0.87 | 1.48 | 0.367 | 0.344 |
| rs1005230 | CC | 296 | 27% | 301 | 29% | 1.00 |  |  |  |  |
| -2487C>T | CT | 541 | 50% | 496 | 48% | 1.16 | 0.94 | 1.43 | 0.165 |  |
|  | TT | 249 | 23% | 236 | 23% | 1.15 | 0.89 | 1.48 | 0.284 | 0.254 |
| rs1570360 | GG | 431 | 48% | 389 | 44% | 1.00 |  |  |  |  |
| -1153A>G | GA | 383 | 43% | 407 | 46% | 0.91 | 0.74 | 1.12 | 0.394 |  |
|  | AA | 78 | 9% | 82 | 9% | 0.91 | 0.64 | 1.29 | 0.584 | 0.398 |
| rs833061 | TT | 237 | 27% | 243 | 28% | 1.00 |  |  |  |  |
| -1497C>T | TC | 434 | 49% | 432 | 49% | 1.08 | 0.85 | 1.36 | 0.536 |  |
|  | CC | 216 | 24% | 198 | 23% | 1.23 | 0.93 | 1.62 | 0.147 | 0.151 |
| rs2010963 | GG | 388 | 44% | 387 | 44% | 1.00 |  |  |  |  |
| Ex1+405C>G | GC | 395 | 45% | 396 | 45% | 0.95 | 0.78 | 1.18 | 0.665 |  |
|  | CC | 98 | 11% | 93 | 11% | 1.01 | 0.72 | 1.40 | 0.963 | 0.864 |
| rs25648 | CC | 746 | 69% | 752 | 73% | 1.00 |  |  |  |  |
| Ex1-73C>T (5'UTR) | CT | 296 | 27% | 271 | 26% | 1.12 | 0.91 | 1.37 | 0.290 |  |
|  | TT | 43 | 4% | 8 | 1% | 5.11 | 2.33 | 11.20 | 0.000045 | 0.0016 |
| rs833067 | TT | 241 | 27% | 243 | 28% | 1.00 |  |  |  |  |
| IVS1+940C>T | TC | 436 | 49% | 428 | 49% | 1.07 | 0.85 | 1.35 | 0.560 |  |
|  | CC | 208 | 24% | 200 | 23% | 1.15 | 0.87 | 1.52 | 0.317 | 0.316 |
| rs3025042 | GG | 879 | 98% | 859 | 98% | 1.00 |  |  |  |  |
| IVS1-627G>T | GT | 16 | 2% | 18 | 2% | 0.85 | 0.42 | 1.72 | 0.642 |  |
|  | TT | 0 |  | 0 |  |  |  |  |  |  |
| rs833068 | GG | 385 | 43% | 382 | 44% | 1.00 |  |  |  |  |
| IVS2+398G>A | AG | 407 | 46% | 397 | 45% | 0.98 | 0.79 | 1.20 | 0.821 |  |
|  | AA | 100 | 11% | 97 | 11% | 0.98 | 0.70 | 1.35 | 0.886 | 0.832 |
| rs3024994 | CC | 837 | 92% | 783 | 89% | 1.00 |  |  |  |  |
| IVS2+1378C>T | CT | 69 | 8% | 96 | 11% | 0.65 | 0.46 | 0.91 | 0.012 |  |
|  | TT | 0 | 0% | 2 | 0% |  |  |  |  |  |
| rs735286 | CC | 431 | 48% | 429 | 49% | 1.00 |  |  |  |  |
| IVS2-585C>T | CT | 377 | 42% | 367 | 42% | 0.98 | 0.80 | 1.21 | 0.879 |  |
|  | TT | 81 | 9% | 76 | 9% | 1.04 | 0.73 | 1.48 | 0.838 | 0.951 |
| rs3024998 | CC | 399 | 45% | 392 | 45% | 1.00 |  |  |  |  |
| IVS3+175C>T | CT | 391 | 44% | 390 | 45% | 0.95 | 0.77 | 1.17 | 0.626 |  |
|  | TT | 98 | 11% | 93 | 11% | 0.97 | 0.70 | 1.35 | 0.852 | 0.711 |
| rs3025000 | CC | 432 | 49% | 437 | 50% | 1.00 |  |  |  |  |
| IVS3-28C>T | CT | 377 | 43% | 361 | 41% | 1.01 | 0.83 | 1.25 | 0.889 |  |
|  | TT | 78 | 9% | 76 | 9% | 1.00 | 0.70 | 1.43 | 0.998 | 0.938 |
| rs3025006 | CC | 346 | 39% | 322 | 37% | 1.00 |  |  |  |  |
| IVS5+593C>T | CT | 408 | 46% | 412 | 47% | 0.88 | 0.71 | 1.09 | 0.250 |  |
|  | TT | 132 | 15% | 137 | 16% | 0.85 | 0.63 | 1.14 | 0.280 | 0.205 |
| rs3025030 | GG | 708 | 80% | 669 | 77% | 1.00 |  |  |  |  |
| IVS7+763G>C | GC | 165 | 19% | 199 | 23% | 0.81 | 0.64 | 1.04 | 0.094 |  |
|  | CC | 10 | 1% | 6 | 1% | 1.75 | 0.61 | 5.04 | 0.299 | 0.274 |
| rs3025033 | AA | 668 | 75% | 631 | 72% | 1.00 |  |  |  |  |
| IVS7-1203A>G | AG | 203 | 23% | 230 | 26% | 0.86 | 0.68 | 1.08 | 0.183 |  |
|  | GG | 17 | 2% | 11 | 1% | 1.58 | 0.71 | 3.51 | 0.265 | 0.530 |
| rs3025035 | CC | 707 | 79% | 706 | 80% | 1.00 |  |  |  |  |
| IVS7-919C>T | CT | 170 | 19% | 163 | 19% | 0.97 | 0.76 | 1.25 | 0.836 |  |
|  | TT | 17 | 2% | 11 | 1% | 1.58 | 0.70 | 3.58 | 0.269 | 0.708 |
| rs3025036 | CC | 396 | 44% | 381 | 44% | 1.00 |  |  |  |  |
| IVS7-609G>C | CG | 380 | 43% | 410 | 47% | 0.89 | 0.72 | 1.10 | 0.277 |  |
|  | GG | 115 | 13% | 82 | 9% | 1.33 | 0.96 | 1.85 | 0.090 | 0.445 |
| rs998584 | CC | 234 | 26% | 237 | 27% | 1.00 |  |  |  |  |
| 5596bp 3' of STP | CA | 439 | 50% | 450 | 52% | 1.00 | 0.79 | 1.26 | 0.971 |  |
|  | AA | 212 | 24% | 177 | 20% | 1.24 | 0.93 | 1.65 | 0.139 | 0.160 |
| rs3025039 | CC | 852 | 78% | 787 | 76% | 1.00 |  |  |  |  |
| 236bp 3' of STP | CT | 217 | 20% | 235 | 23% | 0.87 | 0.70 | 1.08 | 0.209 |  |
|  | TT | 17 | 2% | 11 | 1% | 1.63 | 0.73 | 3.61 | 0.231 | 0.587 |

*Adjusted for age, region, gender and smoking status.

Note: rs3024989 and rs9367173 are not shown because of low genotypic variation in this population (no variants were observed for rs3024989, and 3 controls and no cases were heterozygote for rs9367173).
