# Supplementary material for: Large-Scale Evaluation of Candidate Genes Identifies Associations between VEGF Polymorphisms and Bladder Cancer Risk
Source: PLoS Genet. 2007 Feb 23;3(2):e29. doi: 10.1371/journal.pgen.0030029 (PMC1802828; doi:10.1371/journal.pgen.0030029)
Supplement: Table S5 — (173 KB DOC). [file pgen.0030029.st005.doc]

Table S5: Modification of the association between selected variants in *VEGF* and bladder cancer risk by age, gender, smoking status, family history of cancer, *NAT2* and *GSTM1* genotypes.

|  |  | Common homozygous | | Heterozygous | | Variant homozygous | | Per variant allele | | | | P Inter* |
| --- | --- | --- | --- | --- | --- | --- | --- | --- | --- | --- | --- | --- |
| SNP | Categories | Cases | Controls | Cases | Controls | Cases | Controls | OR* | 95% CI | | |  |
|  | ***Age group, years*** |  |  |  |  |  |  |  |  |  |  |  |
| rs833052 | > 60 | 510 | 482 | 158 | 127 | 16 | 7 | 1.29 | 1.01 | - | 1.64 |  |
|  | ≤ 60 | 167 | 205 | 51 | 62 | 3 | 1 | 1.01 | 0.67 | - | 1.53 | 0.321 |
| rs1109324 | > 60 | 463 | 447 | 192 | 157 | 18 | 6 | 1.27 | 1.01 | - | 1.60 |  |
|  | ≤ 60 | 160 | 198 | 51 | 64 | 7 | 4 | 1.11 | 0.76 | - | 1.62 | 0.553 |
| rs1547651 | > 60 | 460 | 442 | 200 | 165 | 18 | 6 | 1.27 | 1.01 | - | 1.59 |  |
|  | ≤ 60 | 159 | 196 | 51 | 66 | 9 | 3 | 1.17 | 0.81 | - | 1.69 | 0.707 |
| rs25648 | > 60 | 556 | 530 | 229 | 186 | 34 | 5 | 1.44 | 1.17 | - | 1.78 |  |
|  | ≤ 60 | 190 | 222 | 67 | 85 | 9 | 3 | 1.06 | 0.75 | - | 1.49 | 0.126 |
| rs3024994 | > 60 | 634 | 546 | 51 | 67 | 0 | 1 | 0.63 | 0.43 | - | 0.94 |  |
|  | ≤ 60 | 203 | 237 | 18 | 29 | 0 | 1 | 0.61 | 0.33 | - | 1.13 | 0.916 |
|  | ***Gender*** |  |  |  |  |  |  |  |  |  |  |  |
| rs833052 | Female | 77 | 80 | 38 | 24 | 3 | 1 | 1.78 | 1.02 | - | 3.12 |  |
|  | Male | 600 | 607 | 171 | 165 | 16 | 7 | 1.14 | 0.91 | - | 1.43 | 0.148 |
| rs1109324 | Female | 71 | 74 | 41 | 27 | 3 | 2 | 1.48 | 0.88 | - | 2.51 |  |
|  | Male | 552 | 571 | 202 | 194 | 22 | 8 | 1.20 | 0.97 | - | 1.49 | 0.465 |
| rs1547651 | Female | 72 | 73 | 41 | 28 | 4 | 2 | 1.48 | 0.88 | - | 2.48 |  |
|  | Male | 547 | 565 | 210 | 203 | 23 | 7 | 1.21 | 0.98 | - | 1.49 | 0.479 |
| rs25648 | Female | 84 | 93 | 44 | 31 | 5 | 1 | 1.78 | 1.08 | - | 2.92 |  |
|  | Male | 662 | 659 | 252 | 240 | 38 | 7 | 1.27 | 1.05 | - | 1.54 | 0.217 |
| rs3024994 | Female | 109 | 89 | 11 | 15 | 0 | 0 | 0.52 | 0.22 | - | 1.23 |  |
|  | Male | 728 | 694 | 58 | 81 | 0 | 2 | 0.64 | 0.45 | - | 0.92 | 0.659 |
|  | ***Smoking status*** |  |  |  |  |  |  |  |  |  |  |  |
| rs833052 | Never | 88 | 196 | 36 | 56 | 3 | 2 | 1.41 | 0.88 | - | 2.24 |  |
|  | Former | 267 | 257 | 81 | 65 | 8 | 5 | 1.21 | 0.88 | - | 1.67 | 0.600 |
|  | Current | 294 | 182 | 85 | 53 | 6 | 0 | 1.15 | 0.79 | - | 1.66 | 0.503 |
| rs1109324 | Never | 84 | 180 | 39 | 67 | 3 | 4 | 1.18 | 0.76 | - | 1.84 |  |
|  | Former | 248 | 249 | 93 | 70 | 10 | 3 | 1.44 | 1.05 | - | 1.98 | 0.481 |
|  | Current | 267 | 165 | 100 | 68 | 11 | 2 | 1.06 | 0.77 | - | 1.47 | 0.692 |
| rs1547651 | Never | 84 | 177 | 39 | 70 | 4 | 3 | 1.19 | 0.77 | - | 1.84 |  |
|  | Former | 243 | 247 | 100 | 75 | 9 | 2 | 1.48 | 1.07 | - | 2.03 | 0.432 |
|  | Current | 267 | 166 | 101 | 66 | 13 | 3 | 1.10 | 0.80 | - | 1.51 | 0.777 |
| rs25648 | Never | 98 | 211 | 44 | 84 | 5 | 2 | 1.29 | 0.86 | - | 1.93 |  |
|  | Former | 291 | 294 | 117 | 80 | 16 | 4 | 1.60 | 1.20 | - | 2.14 | 0.388 |
|  | Current | 327 | 187 | 121 | 84 | 21 | 1 | 1.12 | 0.84 | - | 1.49 | 0.584 |
| rs3024994 | Never | 118 | 223 | 10 | 28 | 0 | 0 | 0.64 | 0.29 | - | 1.40 |  |
|  | Former | 334 | 286 | 22 | 38 | 0 | 1 | 0.43 | 0.25 | - | 0.75 | 0.434 |
|  | Current | 350 | 212 | 34 | 22 | 0 | 1 | 0.90 | 0.52 | - | 1.56 | 0.473 |
|  | ***Family history***  ***of cancer*** | |  |  |  |  |  |  |  |  |  |  |
| rs833052 | No | 389 | 433 | 121 | 118 | 8 | 5 | 1.25 | 0.95 | - | 1.64 |  |
|  | Yes | 256 | 238 | 81 | 69 | 10 | 3 | 1.21 | 0.87 | - | 1.68 | 0.887 |
| rs1109324 | No | 364 | 392 | 138 | 149 | 10 | 8 | 1.06 | 0.82 | - | 1.36 |  |
|  | Yes | 234 | 240 | 92 | 68 | 15 | 1 | 1.65 | 1.19 | - | 2.30 | 0.035 |
| rs1547651 | No | 360 | 388 | 143 | 156 | 10 | 8 | 1.06 | 0.82 | - | 1.36 |  |
|  | Yes | 234 | 237 | 94 | 71 | 17 | 1 | 1.64 | 1.19 | - | 2.26 | 0.036 |
| rs25648 | No | 435 | 469 | 172 | 188 | 23 | 8 | 1.19 | 0.95 | - | 1.48 |  |
|  | Yes | 286 | 277 | 111 | 79 | 18 | 0 | 1.70 | 1.24 | - | 2.31 | 0.066 |
| rs3024994 | No | 481 | 494 | 37 | 60 | 0 | 2 | 0.60 | 0.39 | - | 0.92 |  |
|  | Yes | 319 | 275 | 30 | 34 | 0 | 0 | 0.77 | 0.45 | - | 1.31 | 0.481 |
|  | ***NAT*2 genotype** |  |  |  |  |  |  |  |  |  |  |  |
| rs833052 | Rapid | 240 | 310 | 76 | 73 | 9 | 5 | 1.39 | 1.00 | - | 1.92 |  |
|  | Slow | 430 | 370 | 131 | 115 | 10 | 3 | 1.10 | 0.84 | - | 1.45 | 0.293 |
| rs1109324 | Rapid | 221 | 293 | 87 | 89 | 12 | 4 | 1.35 | 0.99 | - | 1.84 |  |
|  | Slow | 397 | 347 | 152 | 130 | 13 | 5 | 1.16 | 0.90 | - | 1.51 | 0.466 |
| rs1547651 | Rapid | 220 | 292 | 87 | 92 | 14 | 4 | 1.37 | 1.01 | - | 1.85 |  |
|  | Slow | 394 | 341 | 160 | 137 | 13 | 5 | 1.16 | 0.89 | - | 1.49 | 0.401 |
| rs25648 | Rapid | 256 | 332 | 107 | 109 | 19 | 4 | 1.47 | 1.12 | - | 1.94 |  |
|  | Slow | 485 | 415 | 184 | 159 | 24 | 4 | 1.25 | 0.99 | - | 1.57 | 0.362 |
| rs3024994 | Rapid | 299 | 349 | 25 | 40 | 0 | 0 | 0.73 | 0.42 | - | 1.26 |  |
|  | Slow | 529 | 427 | 44 | 55 | 0 | 2 | 0.57 | 0.38 | - | 0.87 | 0.498 |
|  | ***GSTM1***  **genotype** | |  |  |  |  |  |  |  |  |  |  |
| rs833052 | Present | 262 | 330 | 65 | 89 | 5 | 1 | 0.99 | 0.70 | - | 1.40 |  |
|  | Null | 409 | 351 | 141 | 97 | 14 | 7 | 1.34 | 1.02 | - | 1.75 | 0.184 |
| rs1109324 | Present | 245 | 315 | 74 | 100 | 7 | 4 | 1.04 | 0.76 | - | 1.43 |  |
|  | Null | 373 | 325 | 165 | 118 | 18 | 5 | 1.36 | 1.05 | - | 1.76 | 0.204 |
| rs1547651 | Present | 246 | 312 | 75 | 104 | 7 | 4 | 1.00 | 0.73 | - | 1.37 |  |
|  | Null | 368 | 321 | 172 | 124 | 20 | 4 | 1.40 | 1.09 | - | 1.81 | 0.101 |
| rs25648 | Present | 293 | 366 | 86 | 128 | 15 | 4 | 1.04 | 0.79 | - | 1.38 |  |
|  | Null | 448 | 379 | 206 | 141 | 28 | 2 | 1.56 | 1.23 | - | 1.98 | 0.031 |
| rs3024994 | Present | 305 | 375 | 27 | 45 | 0 | 1 | 0.61 | 0.36 | - | 1.02 |  |
|  | Null | 523 | 399 | 42 | 51 | 0 | 1 | 0.64 | 0.41 | - | 0.99 | 0.878 |
|  |  |  |  |  |  |  |  |  |  |  |  |  |

*Odds ratios per variant allele adjusted for age, region, gender and smoking status.

** P for interaction between genotypes and subject characteristics.
